# Formulation and Targeting Efficiency of Cisplatin Engineered Solid Lipid Nanoparticles

**DOI:** 10.4103/0250-474X.41456

**Published:** 2008

**Authors:** R. C. Doijad, F. V. Manvi, D. M. Godhwani, R. Joseph, N. V. Deshmukh

**Affiliations:** Department of Pharmaceutics, K. L. E. S’s College of Pharmacy, JNMC Campus, Nehru Nagar, Belguam-590 010, India

**Keywords:** Cisplatin, solid lipid nanoparticles, *in vivo* tissue distribution

## Abstract

The present study is aimed at the overall improvement in the efficacy, reduced toxicity and enhancement of therapeutic index of cisplatin. Solid lipid nanoparticulate delivery system of cisplatin has been developed by microemulsification method by using stearic acid, soy lecithin 95% and sodium glycolate. The formulations were then characterized with respect to size and its surface morphology, zeta potential, entrapment efficiency, *in vitro* drug release profile, in vivo drug targeting studies and its stability under specific conditions. The formulated solid lipid nanoparticles were oval with a diameter ranging from 250 nm to 500 nm. The lowest entrapment efficiency was found to be 47.59% and highest was found to be 74.53%. The zeta potential was in the range of -9.8 to -11.2 mv. *In vitro* release study was analyzed using various mathematical models. Highest cumulative percent drug release was observed with F-1 (97.22 %) and lowest with F-4 (78.43%) in 16 h. The *in vivo* result of formulated solid lipid nanoparticles of cisplatin reveals that the drug is preferentially targeting to liver followed by brain and lungs.

Research and development in the field of drug delivery systems facilitating site-specific therapy has achieved significant progression. Safe and nontoxic formulation of cytotoxic drug and its site specific delivery at its target-tumor tissue or tumor cells have become the major goal of the research. Solid lipid nanoparticles (SLN) is a novel nanoparticulate systems made from solid lipids and are attracting major attention as novel colloidal drug carrier, which combines the advantages of polymeric nanoparticles, fat emulsions and liposomes simultaneously and avoids some of their disadvantages. Cisplatin is an important class of antitumor agent and is widely used in the treatment of many malignancies including testicular, ovarian, bladder, head, neck and lung. However the clinical use of this drug is limited due to the emergence of intrinsic and acquired resistance and severe side effects such as acute nephrotoxicity and chronic neurotxicity^1^. Solid lipid nanoparticles are colloidal carriers developed as an alternative system to the existing traditional carrier system (emulsion, liposome, polymeric nanoparticles). Polymeric nanoparticles may contain toxic monomers, residue from solvents, and may form toxic degradation product^2^. The SLN consists of biocompatible lipid core and an amphiphilic surfactant at the outer shell. The additional advantage of the SLN are the large scale production, protection of the drug against chemical degradation, ease of modulation of the drug release profile and high drug pay load^3^. Intravenous administration of cisplatin produces adverse effects like ototoxicity, renal failure, and loss of hearing, optic neuritis, cerebral blindness, papilloedema, tubular necrosis, focal encephalopathy and cardiac abnormalities. All these adverse effects limit the amount of drug to be given to the patient. Hence, to overcome these inherent drawbacks associated with parenteral drug delivery of cisplatin an attempt is being made to provide an alternative drug delivery system of cisplatin in the form of SLN to reduce the adverse effects and to enhance therapeutic efficacy of the drug.

## MATERIALS AND METHODS

Cisplatin was a gift from Cipla Ltd., Bangalore, and Sun Pharmaceutical Ltd., Vadodara. Stearic acid (biochemistry grade) was purchased from Loba Chem Pvt. Ltd., Mumbai, India. Soy lecithin was purchased from Acros Organics, New Jersey, USA. Sodium glycolate was purchased from Acros Organics, New Jersey, USA. Millipore-Academic series (Elix, Mill-Q) were gift sample from Ranbaxy Pvt Ltd., Gurgaon, New Delhi. All other chemicals purchased from S. D. Fine Chemicals, Mumbai, were of analytical reagent grade.

### Preparation of solid lipid nanoparticles by microemulsification technique:

SLN were prepared from a warm o/w microemulsion containing Stearic acid as internal phase, Soy lecithin as surfactant and sodium glycolate as co-surfactant. Microemulsion of cisplatin were prepared by melting lipid (stearic acid) at 80°, to which cisplatin was added and stirred for 5 min, followed by sonication. To this mixture soy lecithin was added and stirred for 2 min. Aqueous phase containing co-surfactant (sodium glycolate) heated at 80° and added to melted lipid phase with mechanical stirring at 80° for 10-15 min, results in o/w microemulsion. This microemulsion was then added carefully drop wise into ice cold water present in a beaker with continuous stirring. Factors such as rate of addition, distance of needle from the surface of the beaker, rate of stirring were standardized to reduce particle size. In order to obtain optimum microemulsion, the needle was placed 4 cm from the surface of the water and mixture stirred at 3000 rpm. The SLN dispersion was further stirred for 3 h after the complete addition of microemulsion. After completion of stirring, the SLN dispersion was subjected to ultra sonication for a period of 10 min^4^.

### Physical evaluation of solid lipid nanoparticles:

Average particle size and surface morphology of SLN were evaluated under scanning electron microscopy (Jeol JSM-T330A). A brass specimen stub were used for mounting the sample and wet solvent paint was applied on the stub, while the paint was wet the pellets were placed on each stub and allowed to dry^5^. Then the sample was observed under scanning electron microscope.

For the drug content assay, the freeze-dried, 10 mg of cisplatin-loaded SLNs were dissolved in ethanol under water bath at 65^°^ for 30 min and then cooled to room temperature to precipitate the lipid. The drug content in the supernatant after centrifugation (1252 × g for 15 min) was measured by UV/Vis spectrophotometer (UV-1201 Shimadzu, Japan) at 210 nm^6^. The drug content in the SLN was calculated using the Eqn., Drug content = (Analyzed weight of drug in SLN/ Theoretical weight of drug loaded in system) × 100. Zeta potential was measured by using zeta potentiometer (Zeta+3 meter, USA). Samples were diluted with KCl (0.1 mM) and placed in electrophoretic cell where the electric field of 15.2 V/cm was applied^7^. Each sample was analyzed in triplicate.

### Evaluation of *in vitro* release:

Solid lipid nanoparticles, equivalent to 10 mg of cisplatin were weighed and transferred into a conical flask containing 50 ml of phosphate buffer saline (PBS pH 7.4) Then the flask was kept in a metabolic shaker and the shaker was adjusted to 50 horizontal shakes per min at 37±0.5°. One ml aliquot of release medium were withdrawn at time intervals of 1, 2, 4, 8, 16, 24 h and replaced by the same volume of PBS^8^. Samples were filtered through 0.45 μm membrane filter (Elix, Mill-Q) and the filtrate was diluted suitably with PBS and estimated by UV/Vis spectrophotometer at 210 nm.

### *In vivo* tissue distribution:

This study was carried out after obtaining the due permission for conduction of experiments from relevant ethics committee (K. L. E. S’s College of Pharmacy, Belgaum) which is registered for “Teaching and Research on Animals” by committee for the purpose of control and supervision of experiments on animal, Chennai (Registration number 221/CPCSEA). It is performed to compare the targeting efficiency of drug-loaded solid lipid nanoparticles with that of free drug in terms of percentage increase in targeting to various organs of reticuloendothelial system like liver, lungs, spleen, kidney, heart and brain^9^.

Nine healthy adult Wistar rats weighing 200-250 g were selected, a constant day and night cycle was maintained and they were fasted for 12 h. The animals were divided into 3 groups, each containing 3 rats. Group I received solid lipid nanoparticles equivalent to 607.5 μg of cisplatin intravenously in the tail vein after redispersing them in sterile phosphate buffer saline solution, F3 (optimized) batch were selected for the study. Group-II rats received 607.5 μg of pure cisplatin intravenously. Group-III rats were treated as solvent control and were injected intravenously with sterile phosphate buffer saline solution.

After 3 h the rats were sacrificed and their liver, lungs, spleen, kidney, heart and brain were isolated. The individual organs of each rat were homogenized separately by using a tissue homogenizer with 5ml of ethanol and the homogenate was centrifuged at 17609 × g for 30 min. The supernatant was collected and filtered through 0.45 μ filters and analyzed spectrophotometrically after suitable dilution with phosphate buffer saline at 210 nm^10^.

### Stability studies:

The purpose of stability testing is to provide evidence on how the quality of a drug substance or drug product varies with time under the influence of variety of environmental conditions such as temperature, humidity and light. The best batch of cisplatin loaded solid lipid nanoparticles were tested for their stability. All the preparations were divided into 3 sets and were stored at 4±2° in refrigerator, 25±2°/60%±5% RH and 37±2°/65%±5% RH in humidity control oven (Ginkya IM 3500 Series). After 3 mo, the drug content and *in vitro* release of all the formulation was determined by the method discussed previously^11^.

## RESULTS AND DISCUSSION

To ensure the compatibility of the drug with polymers preformulation studies were done using IR spectrum recorded on Impact-410 Nicolet, FT-IR USA, by preparing KBr disk. The IR peaks of cisplatin with the polymers resemble almost same structural peaks of pure cisplatin indicating the compatibility between the drug and polymers.

**Fig. 1:**
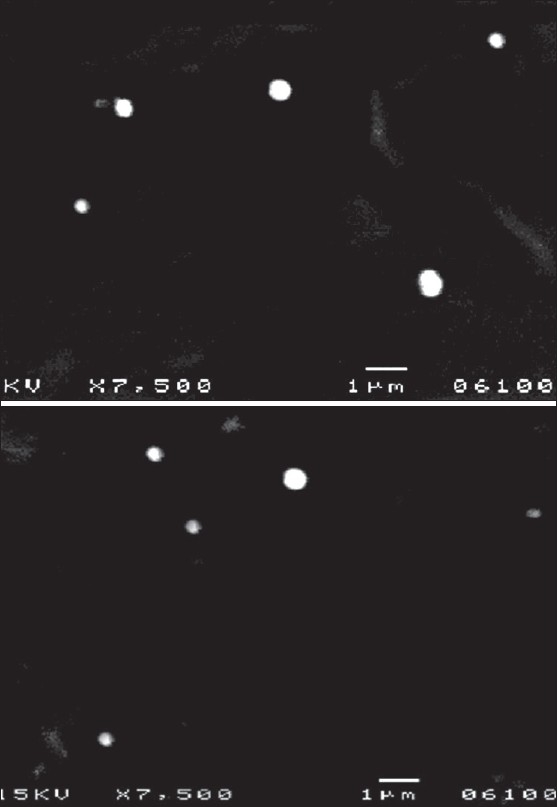
Scanning electron microscope photographs of solid lipid nanoparticles.

Cisplatin loaded solid lipid nanoparticles were in the range of 250-500 nm and the surface morphology of the particle revealed that the particle were spherical in shape and discrete (fig. 1). Scanning electron microscopy also reveled that the particle size increases with the increase in the lipid concentration. Results of zeta potential showed plateau of slight stability and few aggregations.

The amount of drug bound to the SLN was found to increase with the increase in the stearic acid concentration and this may be due to the higher intactness of the lipid. The maximum drug entrapment was found with F4 (74.53%) and lowest entrapment with F1 (47.59%) (Table 1). It was also found that, the increase in lipid concentration affects the *in vitro* release kinetics, higher lipid ratio leads to high initial burst release.

**TABLE 1:** DRUG ENTRAPMENT EFFICIENCY OF CISPLATIN LOADED SOLID LIPID NANOPARTICLES

Formulation	Concentration (μg/ml)	Drug content (μg/10 mg)	% Drug content
F1	48.13±0.223	722±3.357	47.59±0.221
F2	26.53±0.168	398±2.524	60.42±0.383
F3	17.46±0.141	262±2.109	69.18±0.557
F4	11.8±0.066	177±0.994	74.53±0.418

Each value represents mean±SD for (n=3)

**Fig. 2:**
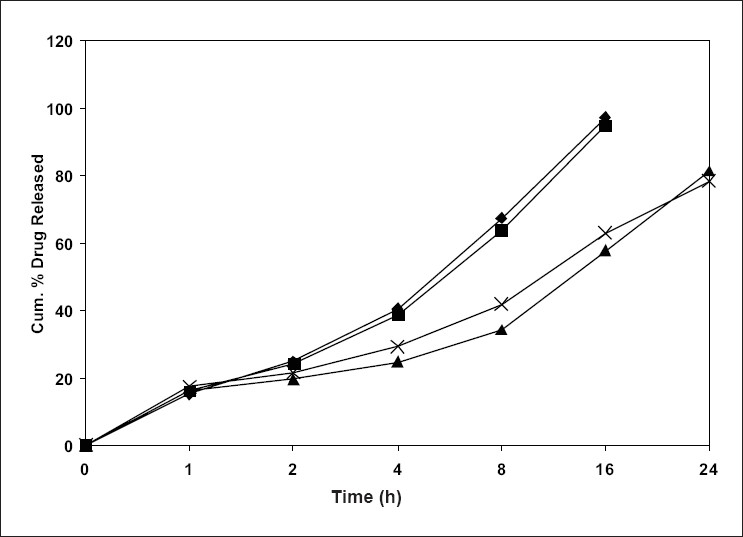
Plot of cumulative % drug released vs time for different formulation of cisplatin solid lipid nanoparticles. *In vitro* release profile (zero order plot) for different formulation of cisplatin solid lipid nanoparticles, formulation F1 (– ♦ –), formulation F2 (– ■ –), formulation F3 (– ▲ –) and formulation F4 (– × –).

The *in vitro* release of all the four batches of SLN (F1 to F4) were carried out, which showed an interesting biphasic release with an initial burst effect in the 1^st^ h drug release was 15.18%, 16.2%, 16.22% and 17.34% for F1, F2, F3 and F4, respectively. This was followed by a prolonged second phase (zero order) release, which may due to diffusion of drug through the polymer matrix as the lipid erodes slowly. Cumulative percent drug release for F1, F2 after 16 h was 97.22%, 94.88% and for F-3, F-4 after 24 h was 81.39% and 78.43%, respectively, and was 93.93 % in 3 h for pure drug cisplatin (fig. 2).

**Fig. 3:**
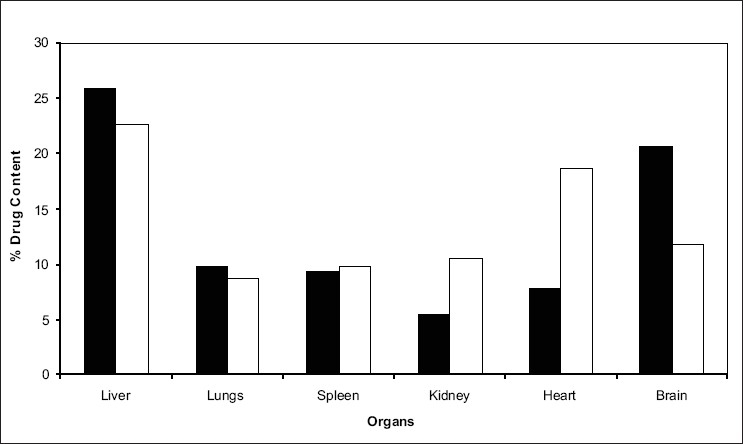
Comparison between amounts of drug distributed from formulation F3 after i.v. administration. Optimized formulation F3 is denoted by (■) and pure drug is denoted by (□).

Formulation (F3) with optimal particle size and satisfactory *in-vitro* release was selected for *in vivo* drug targeting studies. The comparison between the amount of drug targeted from SLN and pure drug in various organs are presented in (fig. 3). The average targeting efficiency of drug loaded SLN was found to be 25.93% of the injected dose in liver, 9.79% in lungs, 9.33% in spleen and 5.4% in kidney, 7.78% in heart, and 20.6% in brain, whereas accumulation of pure drug was 22.65% in liver, 8.72% in lungs, 9.72% in spleen, 10.57% in kidneys, 18.66% in heart, and 11.7% in brain of the injected dose. These results reveal that the drug loaded solid lipid nanoparticles showed preferential drug targeting to liver followed by brain, lungs, spleen, heart and kidneys. Compared to pure drug, higher concentration of drug was targeted to the organs like liver and brain after administering the dose in the form of solid lipid nanoparticles. Higher drug targeting in brain and liver showed that the increased lipophilicity of cisplatin loaded SLN, played an important role in increasing the brain targeting efficiency.

Stability studies revealed that, there is no much reduction in drug content after storage for 3 mo at 4±2° in refrigerator, 25±2°/60%±5% RH and 37±2°/65%±5% RH in humidity control oven (Ginkya IM 3500 Sieries). *In vitro* release studies were carried out at the end of 3^rd^ mo proved that the formulation stored at 4° showed 83.68% release, the one which stored at 25° showed 85.68% and formulation stored at 37° showed 84.27% release after 24 h. These results indicate that the drug release from the formulation stored at 25° was highest followed by formulation stored at 37° and 4°. On comparing this data with the previous release data of F3, it was observed that there was an overall increase in the drug release. These results may be attributed to erosion of lipid particles to some extent during storage. Thus it can be concluded that 4° is the most suitable condition for storage of cisplatin loaded solid lipid nanoparticles.
